# Evaluation of the Tensile and Puncture Properties of Geotextiles Influenced by Soil Moisture under Freezing Conditions

**DOI:** 10.3390/ma17020376

**Published:** 2024-01-11

**Authors:** Lanjun Liu, Haiku Zhang, Jinhuan Zhu, Shixin Lv, Lulu Liu

**Affiliations:** 1 Shangqiu Institute of Technology, Shangqiu 476000, China; lljtmzy@163.com (L.L.); z2434837747@163.com (J.Z.); 2 Fuyuan Ding Installation Engineering Company Limited, Shangqiu 476000, China; 15729203788@163.com; 3 School of Highway, Chang’an University, Xi’an 710064, China; lsx1052332819@163.com; 4 School of Mechanics and Civil Engineering, China University of Mining and Technology, Xuzhou 221116, China

**Keywords:** geotextiles, tensile and puncture properties, failure mechanism, low temperature, moisture content

## Abstract

Freezing conditions under different humidity will influence the mechanical properties of geotextiles, leading to the gradual fracture of geotextiles. It brings hidden danger to the whole isolation, reinforcement and protection of rock and soil. It is particularly important to study the tensile and puncture properties of geotextiles considering low temperature and moisture content. In this paper, a series of tensile and puncture tests of geotextiles are performed under different low temperatures (0, −3, −6, −9, and −12 °C) and at different moisture content levels (0, 5, 10, 30, 50, and 80%). From the microscopic perspective, the failure mechanism considering the low temperature and moisture content was explained comprehensively. Experimental results indicate that with a decrease in freezing temperature, the tensile strength of geotextiles increases as a parabolic function while the elongation at failure decreases as an exponential function. Additionally, the puncture strength of geotextiles presented a parabolic increase with the decreasing temperature. Under the freezing temperature environment, the higher moisture content of geotextiles can generate a higher puncture strength increment. This research contributes to a more comprehensive understanding of the tensile and puncture properties of geotextile materials considering low temperature and moisture content. It can provide important guidance for the design of slopes, the reinforcement of earthen dams, and roadbed reinforcement with geotextiles in cold regions.

## 1. Introduction

Geotextile materials have emerged as a cornerstone in the realm of civil engineering, particularly revered for their multifaceted utility in projects requiring enduring resilience and adaptability [1,2,3]. Their applications span an impressive range of engineering challenges, from fortifying roadbeds and buttressing slopes to facilitating highway upkeep and use in the undergirding of tunnel construction. The widespread adoption of geotextiles is attributable to a unique combination of physical properties: their lightness simplifies handling and transportation; excellent permeability allows for efficient water drainage while averting waterlogging; remarkable tensile strength ensures durability under strain; and resistance to high temperatures and corrosion guarantees longevity in harsh environments [4]. These characteristics are not merely theoretical assertions but are well documented in a number of scientific studies [5,6,7]. In the specific context of geotechnical engineering, geotextiles serve a pivotal role in safeguarding the integrity of geological substrates. When applied, they are subjected to a biaxial tension state, a direct consequence of the load distribution from above and lateral constraints. However, this state of stress renders them vulnerable to potential damage. For instance, when encased materials shift, generating stresses that exceed the fabric’s tensile and penetration thresholds, or when they encounter sub-surface sharp objects like gravel or stones, the integrity of the geotextiles can be compromised [8,9]. The occurrence of such punctures not only undermines the material’s protective capabilities but also poses a latent risk to the structural stability of the engineering project. Given these risks, the study of geotextile durability—specifically, tensile and puncture resistance—is of paramount importance [10]. Delving into the properties that govern their performance under stress can yield valuable insights, leading to the development of more robust geotextile variants. This knowledge is essential for advancing the reliability of geotextile applications in critical infrastructure, thereby ensuring that these materials continue to perform their intended protective functions throughout the lifespan of the engineering projects they support [11].

Geotextiles, when deployed as a layer embedded within the soil, are highly susceptible to the prevailing environmental conditions. This susceptibility becomes notably significant in colder climates where sub-zero temperatures are common. In such environments, the drop in temperature leads to the formation of ice within the soil matrix, which can severely impact the geotextiles by altering their structural characteristics. The presence of ice can drastically affect tensile strength, which is the capacity of the geotextile to withstand pulling forces, and puncture resistance, which is the ability to resist breaking or tearing upon the application of a sharp force [12]. Considering the pivotal role that geotextiles play as a reinforcing and stabilizing element in construction, it becomes imperative to conduct an in-depth analysis of their performance. This analysis must focus on understanding how low-temperature scenarios, especially when coupled with moisture, can influence the material properties and integrity of geotextiles. By doing so, it is possible to ensure the reliability and durability of construction projects that incorporate these materials in challenging weather conditions.

As for geotextiles, the investigations in the literature mainly focused on evaluating tensile properties [13,14], puncture properties, creep behavior [15], interfacial behavior [16,17], and microstructure [18,19] under ambient temperature conditions.

The effects of high temperature on the behavior of geotextiles have been investigated by several researchers. For example, Kongkitkul et al. indicated that the tensile rupture strength of polypropylene (PP), high-density polyethylene (HDPE), and polyester (PET) geogrids decreased by 9.2%, 26.7%, and 4.5%, respectively, as the temperature increased from 30 to 50 °C [20]. Similarly, Chantachot et al. also revealed that an increasing ambient temperature could reduce the rupture tensile strength and elastic stiffness of geogrids while improving creep strain during sustained loading, which was consistent with the previous results [21,22].

So many studies have been conducted on the performance of geotextiles working under and above ambient conditions, while limited knowledge is available on the behavior of geotextiles working in freezing temperatures. Allen (1983) reported that a freezing temperature (−12 °C) could reduce the strain at failure value of needle-punched polyester by 59–86% compared with an ambient temperature, while the failure modulus and elongation could reach 60% and 88% of the ambient temperature, respectively. Nevertheless, there is a lack of in-depth understanding regarding the tensile and puncture properties of geotextiles considering low-temperature and wet conditions, especially the failure mechanism of geotextiles due to the formation of ice under freezing conditions.

In general, considering the influence of low temperature and moisture content, the tensile and puncture properties and failure mechanism of geotextiles need further study. In light of this, a series of tensile and puncture tests were performed in this study. The effect of different freezing temperatures and moisture content on tensile strength, elongations at failure, and puncture strength are analyzed in detail. Furthermore, the failure mechanism considering low temperature and moisture content are explained comprehensively.

## 2. Experimental Content

### 2.1. Materials

In this comprehensive study, the geotextile samples under examination were meticulously sourced from an active construction zone located along the Bao-Han highway, specifically pinpointed at a marker of K36 + 300 within the Baoji region of Shaanxi Province, China. The production process of these geotextiles involves a mechanical interlacing technique that intricately combines multiple layers of diverse fibers, all aligned in the direction of the machinery to ensure uniformity and structural integrity. The resulting geotextile product boasts an average thickness measurement of 2.8 mm, which contributes to its substantial feel and effectiveness in application. Additionally, it presents a significant mass per unit area, registering at 400 g/m^2^, which is indicative of its robustness and suitability for the demanding tasks it is designed for in civil engineering projects.

In this study, we have chosen polypropylene and polyester fibers as the primary materials for the geotextile. After careful consideration, we decided on a 50/50 ratio of polypropylene to polyester fibers. This ratio balances the mechanical strength and durability of the materials, making them suitable for a variety of civil engineering applications. This decision is based on a comprehensive evaluation of the properties of both fibers, aimed at ensuring the geotextile exhibits optimal performance characteristics.

### 2.2. Experimental Apparatus

A tensile testing machine was used to test the tensile strength and elongations. The machine has a variety of stress and strain control modes, with a load rate of 0.5–500 mm/min, a maximum load level of 100 kN, and load control precision of ±0.1% [23].

A puncture testing machine was applied to measure puncture strength with a strength test range of 0–20 kN, a puncture down speed of 100 mm/min, and an effective maximum dynamic range of 90 mm [24].

A freeze–thaw box was used to simulate the ambient and freezing temperatures for geotextile specimens. The adjustable temperature ranged from 80 to −20 °C and control accuracy was ±0.1 °C.

In the experimental section of our paper, particularly in the subsection discussing SEM images and characterization techniques, the following paragraph is added: ‘To comprehensively characterize the geotextile samples, Scanning Electron Microscopy (SEM) was employed for microstructural analysis. The specific SEM equipment utilized was the JEOL JSM-7800F Prime, manufactured by JEOL Ltd., Tokyo, Japan. This high-resolution field emission SEM is renowned for its exceptional imaging capabilities, with technical specifications including magnification up to 200,000× and superior resolution. The sample preparation process involved coating and appropriate drying of the geotextile samples, ensuring high-quality images for SEM analysis. These detailed equipment specifications and sample preparation steps are crucial for understanding the reproducibility and accuracy of the experimental results.

In this study, we employed the Instron Universal Testing Machine, model 5969, produced at Nanjing Instrument Factory in Jiangsu Province, China, for the testing of tensile strength and elongation. The Instron 5969 is widely recognized and used in the field of material testing due to its high precision in control and its extensive range in measuring stress and strain. Its broad range of stress and strain control modes, along with a maximum load level of 100 kN and load control precision of ±0.1%, make it an ideal choice for our study. The high accuracy and reliability of this equipment are crucial for ensuring the precision of our geotextile material performance tests.

The tensile tests were conducted in accordance with the ASTM D4595 [23] standard, while the puncture tests followed the guidelines of ASTM D6241 [24].

### 2.3. Specimen Preparation

In preparation for a comprehensive study on the effects of extreme cold on geotextiles, a vital material in construction, specimens were meticulously prepared to mimic the harsh winter conditions of Shaanxi Province. During Shaanxi’s coldest months, temperatures can plummet to as low as −10 °C, posing a significant challenge to the durability and performance of construction materials. In order to understand how these cold temperatures affect the mechanical properties of geotextiles, researchers conducted a series of humidity tests on the samples, particularly at 0%, 5%, 15%, 30%, 50%, and 80% humidity. The methodology was meticulous, with each geotextile first equilibrated to its specific moisture content by being placed in a controlled curing chamber maintained at a constant 20 ± 2 °C at 95% relative humidity for 72 h. Following this, they were then exposed to a freeze–thaw cycle in a box set at varying freezing temperatures of −3, −6, −9, and −12 °C for 12 h to simulate the harsh winter conditions. Upon removal from these severe freezing conditions, the geotextile samples were immediately subjected to mechanical testing, specifically tensile and puncture resistance tests. This quick transition from the freeze–thaw box to testing was critical to minimize any potential alterations in the samples due to temperature changes, which could compromise the integrity of the data. The production of the geotextile employed a mechanical interlacing technique, effectively combining polypropylene and polyester fibers in a layered structure, ensuring product uniformity and structural integrity. Additionally, vinylon was incorporated into the fiber composition to enhance the water and heat resistance of the geotextile in specific environments. The inclusion of vinylon is based on its unique performance attributes, allowing the geotextile to be suitable for a wider range of applications, especially in projects demanding high water and heat resistance. In addition, five parallel specimens were prepared for each test scheme. Under the given moisture content and test temperature, five parallel specimens were tested, and the average values were taken as test results to reduce fluctuation errors in test data.

## 3. Results and Discussion

### 3.1. Tensile Properties

#### 3.1.1. Tensile Strength

**Figure 1:**
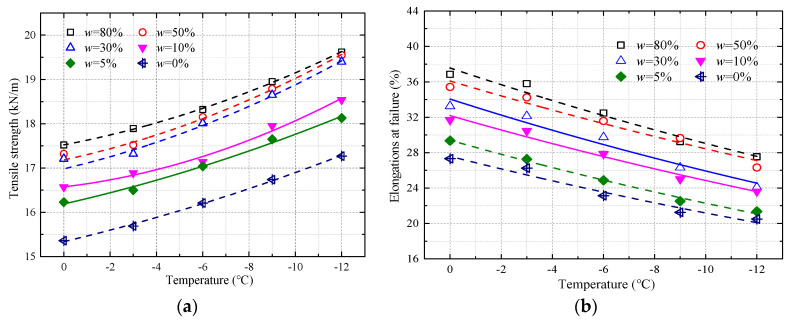
Tensile strength and elongations at failure versus temperature under different moisture content in geotextiles: (**a**) tensile strength versus temperature; (**b**) elongations at failure versus temperature.

Figure 1a illustrates the relationship between tensile strength and temperature across various levels of moisture content within geotextile materials. From the graph, a clear pattern emerges where the tensile strength appears to elevate in a parabolic manner as the temperature drops below the freezing point. This trend is consistent across the range of temperatures studied, suggesting a robust link between colder conditions and the mechanical properties of the materials in question. It is noteworthy that, at any specific freezing temperature, the tensile strength is positively correlated with the moisture content; in other words, the more moisture present within the geotextile fibers, the higher the tensile strength recorded. This enhancement of tensile strength in wetter conditions, particularly under sub-zero temperatures, implies that the presence of water molecules may be playing a crucial role in reinforcing the structural integrity of the geotextiles when they are subjected to freezing conditions. The data may suggest that the formation of ice crystals within the fibers could be contributing to this strengthening effect, as the freezing process possibly facilitates additional bonding within the material structure, leading to an increase in tensile resistance. This phenomenon highlights a potential avenue for improving the performance of geotextile materials in cold and wet environments by optimizing moisture content to harness this strength-augmenting effect.

In the freezing temperature environment, the molecular activity inside the geotextile is hindered, and the intermolecular force increases. Therefore, a greater external load is required to overcome the increased interaction between molecular bonds and cause the directional arrangement between molecular bonds to move [18]. For a given temperature, the change rate of tensile strength under different moisture content (5–80%) is between 13.0 and 14.67% compared with that under dry conditions. For a given moisture content, the change rate of tensile strength at different freezing temperatures (−3–12 °C) is 11.71–13.87% compared with that under the temperature of 0 °C. Therefore, the effect of freezing temperature on tensile strength is less than that of moisture content.

Three function models (the exponential, linear, and parabolic functions) were used to fit the test data. The parabolic function can present the best fitted effect (Table 1), and the fitted equation of the tensile strength and freezing temperature of geotextiles is as follows:

(1) $$\mathrm{Pt}=aT^{2}+bT+c$$

where Pt represents the tensile strength, kN/m; T represents the temperature, °C; and a, b, and c represent the fitted coefficients.

#### 3.1.2. Elongations at Failure

From 0 to −12 °C, the change in elongations at failure for geotextiles with a moisture content of 30% was largest, reaching 34.22%. When moisture content was less than 5%, the influence of moisture content on elongations at failure was decreased gradually compared with higher moisture content. It was noteworthy that, at the same freezing temperature, the wet geotextiles that generated more ice exhibited more brittle behavior than the dry geotextiles, which generated lower elongations at failure. In Figure 1b, a comprehensive analysis showcases the impact of temperature on the stretching capability of geotextiles at the point of rupture when subjected to varying levels of moisture content. The study indicates a clear correlation between the decrease in freezing temperature and the reduced capacity of the geotextiles to elongate upon reaching their failure threshold. This reduction is not arbitrary but follows an exponential relationship with the descending temperatures, highlighting a fundamental change in material behavior in colder climates.

While there is an observable increase in the tensile strength of geotextiles as the temperature drops, it comes at a significant cost to the material’s overall ductility. In environments characterized by a specific moisture content paired with freezing temperatures, the toughness of the geotextile material, or the ability to absorb energy prior to failure, was adversely affected. This transition results in a compromised yield capacity, where the material’s molecular chains relax more swiftly, thus precipitating a marked decrease in the ability of the geotextiles to stretch to their limits without breaking.

This phenomenon was particularly evident when the geotextiles were exposed to a temperature range from 0 to −12 °C with 30% moisture content. Under these conditions, the elongations at failure observed a significant decline, with a recorded decrease of 34.22%. Interestingly, when the moisture content was maintained below 5%, the impact on the elongations at failure began to taper off, especially when compared to geotextiles with higher moisture content. This suggests that moisture content plays a critical role in the material’s performance, particularly under freezing conditions. A notable observation was that geotextiles with higher moisture content, which consequently formed more ice within their structure, tended to exhibit a more brittle nature. This brittleness translated into a lower threshold for elongation at failure compared to their drier counterparts. Such a difference underscores the importance of considering both temperature and moisture content when evaluating the durability and suitability of geotextile materials for use in cold environments, where ice formation can drastically alter their mechanical properties and performance.

Similar to tensile strength, the exponential function model (Table 2) can describe the change tendency of elongations at failure with temperature, and the fitted equation of elongations at failure under freezing temperatures of geotextiles is as follows:

(2) $$L=de^{fT}$$

where L represents the elongations at failure, %, and d, e, and f represent the fitted coefficient.

### 3.2. Puncture Properties

#### 3.2.1. Puncture Strength

**Figure 2:**
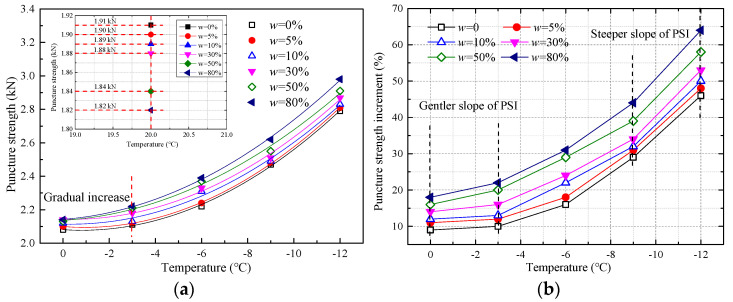
Puncture strength and puncture strength increment versus temperature at different moisture content levels: (**a**) puncture strength versus temperature; (**b**) puncture strength increment versus temperature.

Figure 2a provides a graphical representation of the relationship between the puncture strength of geotextiles and temperature across various moisture levels. From the chart, it becomes evident that as the moisture content in the geotextiles escalates from a completely dry state to a saturation level of 80%, there is only a minor decline in puncture strength, specifically a reduction of 0.09 kilonewtons (kN) at room temperature. This marginal change indicates noteworthy resistance in the material’s puncture strength in relation to moisture alteration. The underlying reason for this resilience can be attributed to the intermolecular friction among the polymer chains constituting the geotextiles, which only moderately diminishes as the moisture content rises. Despite the presence of moisture, the fibers maintain a considerable amount of their interaction, hence preserving the structural integrity of the geotextile.

However, an interesting phenomenon occurs when the geotextiles are subjected to sub-zero temperatures. As the temperature drops below the freezing point, to −3 °C, −6 °C, −9 °C, and then to −12 °C, there is a noticeable incremental increase in the material’s puncture strength, with increases of 0.88 kN, 0.91 kN, 0.94 kN, 1.04 kN, and 1.16 kN, respectively. This trend clearly demonstrates that freezing temperatures act to enhance the puncture strength of the geotextile fabric. This is likely due to the formation of ice within the fabric, which binds the polymer molecules more tightly together, essentially reinforcing the material’s structure against puncture forces.

Notably, the rate at which the puncture strength increases with the decrease in temperature is not constant. In the temperature range from 0 °C to −3 °C, the rate of increase in puncture strength is relatively slower. In contrast, from −3 °C to −12 °C, the rate of increase is more pronounced. The explanation for this might be associated with the stage at which the geotextiles initially have to disrupt the ice’s bond strength before contending with the intermolecular bonding strength of the polymers. As the temperature plunges, the bonding force between the polymer particles intensifies due to decreased molecular activity. Consequently, a greater amount of external force is required to disrupt these bonds, resulting in the amplification of puncture strength. This suggests that geotextiles exhibit an enhanced performance under freezing conditions, making them particularly effective for applications in cold environments where they are subjected to sharp objects or conditions that could potentially cause punctures [25].

In addition, the parabolic function (Table 3) with the best fitted effect for puncture strength and freezing temperature was obtained:

(3) $$P_{p}=gT^{2}+hT+i$$

where Pp represents the puncture strength, kN; T represents the temperature, °C; and g, h, and i represent fitted coefficients. 

#### 3.2.2. Puncture Strength Increment

To describe the effect of low temperature on the puncture strength of geotextiles under different moisture content levels more comprehensively, the concept of the puncture strength increment (PSI) of geotextiles was proposed:

PSI = 100 × (Pp-Pp−20 °C)/Pp−20 °C(4)

where PSI denotes the puncture strength increment, %; Pp denotes the puncture strength at different freezing temperature, kN; and Pp−20 °C denotes the puncture strength at an ambient temperature (20 °C), kN. 

The relationship between the puncture strength increment and temperature at different moisture content levels is plotted in Figure 2b. At temperatures less than −3 °C, there is a gentler slope in PSI, while the steeper slope in PSI occurred when the temperature exceeded −9 °C. The obvious variation in PSI under different moisture content levels can be noted. For example, at a given temperature (−6 °C), the PSI at the moisture content level of 80% was 1.78 times that of the PSI at a moisture content level of 5%. The lower the temperature, the greater the puncture strength increment was. Additionally, under the freezing temperature environment, higher moisture content in geotextiles can generate greater puncture strength increments.

### 3.3. Failure Mechanism Analysis Based on Low Temperature and Moisture Content

The main components of staple needle-punched geotextiles are polyester, polypropylene, and vinylon, each of which has porous spaces [18]. The ‘geotextile matrix’ refers to the intricate network of fibers composing the geotextile fabric. Within this matrix, water molecules primarily situate in the interstices between fibers.

As temperatures drop below freezing, these water molecules freeze, forming ice within these interstitial spaces. This ice formation occurs on a macroscopic scale within the voids between fibers, rather than within the fibers themselves. It is important to note that the ice does not create a continuous matrix akin to that in composite materials but rather fills the gaps between fibers, altering the fabric’s overall structure.

**Figure 3:**
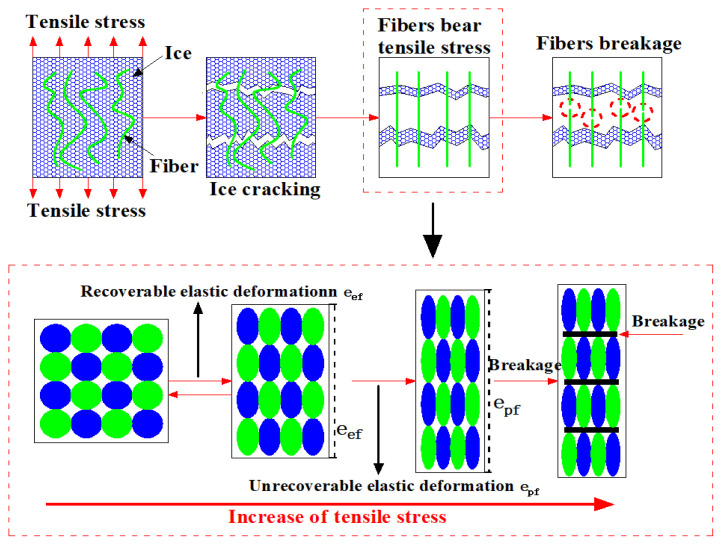
Analysis of the failure mechanism in wet geotextiles at freezing temperatures.

**Figure 4:**
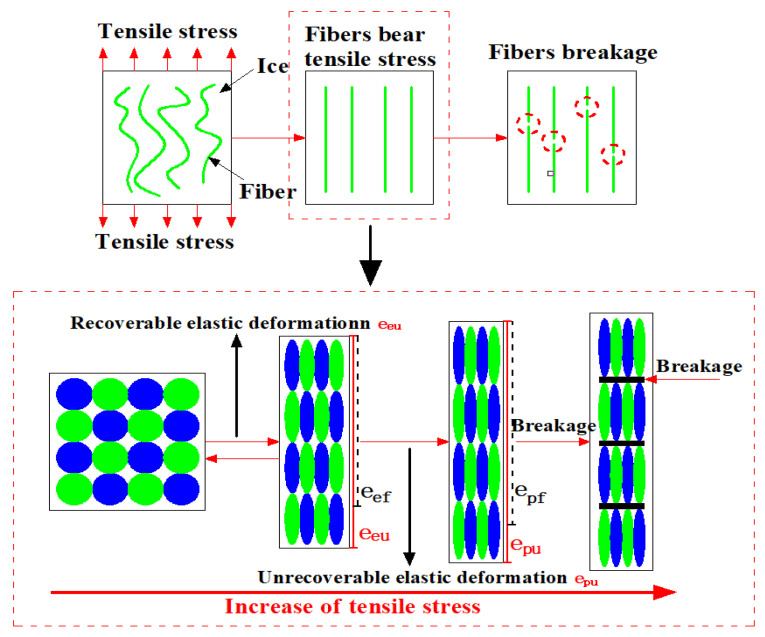
Analysis of the failure mechanism in dry geotextiles at normal temperatures.

The comprehensive analysis of Figure 3 and Figure 4 reveals a critical aspect of material science concerning geotextiles subjected to tensile stresses under freezing conditions. Through polymer molecular bonding, the internal mechanism change chart of geotextiles under freezing temperatures and ambient temperatures is established in Figure 3 and Figure 4, respectively. Among them, e_ef_ and e_pf_ represent the elastic and plastic deformation of geotextiles at freezing temperatures. e_eu_ and e_pu_ denote the elastic and plastic deformation of geotextiles at room temperature.

The formation of ice impacts the mechanical properties of the geotextile by introducing an additional phase within the matrix, which changes the way the material responds to mechanical forces. When subjected to tensile stress, the geotextile now contains both the original fibrous material and the interspersed ice, leading to a composite-like behavior where both components contribute to the overall response of the material to stress. This unique interaction at the macroscopic level, between the geotextile fibers and the ice, is critical to understanding the failure mechanism of geotextiles under freezing conditions. From the failure progress of geotextiles, the puncture failure can be regarded as the tensile failure.

This phenomenon has implications for the mechanical properties of geotextiles. The inherent bonding strength between the polymer chains that make up the geotextile fibers, and that between the ice molecules, forms a dual barrier to material failure. Initially, as the tensile load is applied, it is the ice that resists, with its molecular bonds cleaving under stress. Following this, the geotextile fibers, now interlaced with fragments of shattered ice, begin to bear the load. These ice fragments act almost like a secondary reinforcing agent, creating an additional bonding force that enhances the tensile capacity of the geotextile fibers. This interaction continues until the ultimate tensile strength of the polymer chains is reached and they succumb to the applied force, leading to failure.

To further elaborate on the directional nature of the measurements, our tensile tests were primarily conducted along the principal fiber orientation of the geotextiles, aligning with their structural and manufacturing design. This direction, typically parallel or perpendicular to the roll direction, is crucial for assessing tensile behavior under freezing conditions. The formation of ice in the geotextile matrix and its subsequent impact on tensile strength and failure were thoroughly evaluated in these specific directions. This approach provides insights into how the ice influences the geotextile’s mechanical response when subjected to stress in these key orientations.

**Figure 5:**
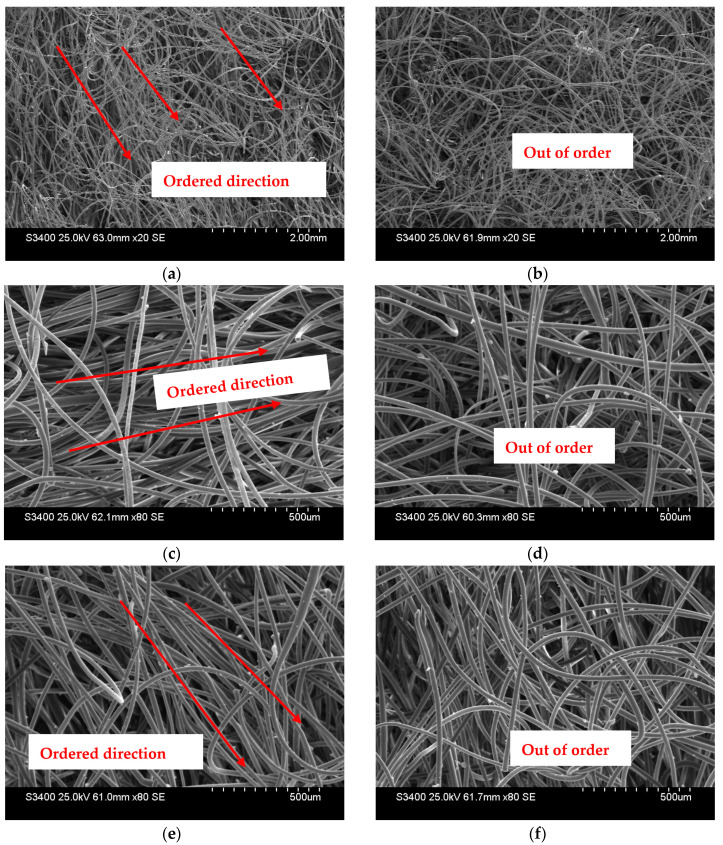
SEM images of geotextiles after tensile failure in dry and wet conditions. (**a**) T = −5 °C, w = 80%, 20 times. (**b**) T = −5 °C, w = 0%, 20 times. (**c**) T = −3 °C, w = 80%, 80 times. (**d**) T = −3 °C, w = 0%, 80 times. (**e**) T = −6 °C, w = 80%, 80 times. (**f**) T = −6 °C, w = 0%, 80 times.

Furthermore, Figure 5a–f provide additional insights into the behavior of geotextiles under these conditions. These figures depict the directional nature of fiber failure in wet geotextiles as compared to those in dry conditions. The ice plays a significant role in this directional failure, suggesting that the presence of frozen water within the fabric influences the alignment and subsequent breakage pattern of the fibers. The SEM analysis presented in these figures, particularly between Figure 5c,e, illustrates a clear correlation between temperature and the orderly nature of the failure. The colder the temperature, the more pronounced and orderly the direction of geotextile failure, underscoring the role of temperature in the mechanical failure process of geotextile fibers.

In conclusion, the interactive effects of temperature, ice formation, and polymer bonding within geotextiles present a complex mechanical behavior under tensile stress. The study presented in these figures contributes significantly to our understanding of the performance and resilience of geotextile materials in harsh, freezing environments, offering valuable insights for the design and application of geotextiles in cold regions.

When the geotextile fiber begins to bear forces, due to the effect of freezing temperature on the geotextile fiber, the elastic deformation $\left(e_{ef}\right)$ at freezing temperatures is less than ${(e}_{eu})$ at ambient temperatures, and the plastic deformation ${(e}_{pf})$ at freezing temperatures is less than ${(e}_{pu})$ at ambient temperatures. In the elastic deformation stage, only recoverable tensile deformation occurs for polymer molecules due to the shorter time period. In the plastic deformation stage, the polymer molecule exhibits irreversible deformation, and only part of the deformation can be restored [25]. In comparison with the ambient temperature, although the tensile strength increases under the freezing temperature, the overall toughness decreases, which enhances brittleness and decreases the yield ability of the geotextile fiber. Consequently, the activity of polymer molecular chains decreases under the influence of low temperature, and the active region gradually shrinks, shortening the elastic deformation and plastic deformation phases [26,27].

## 4. Conclusions and Suggestions

In this study, a series of laboratory tests were conducted to evaluate the tensile and puncture properties of geotextiles considering the effect of various freezing temperatures and moisture content levels. The main conclusions are drawn as follows:

1. The parabolic and exponential function model of tensile strength and elongations at failure with the decreasing temperature of geotextiles was proposed considering different moisture content levels. In addition, the tensile strength and elongations at failure reached the maximum values of 14.67% and 34.22%, respectively, at the moisture content of 30% when the temperature decreased from 0 to −12 °C.
2. The puncture strength of geotextiles presents a parabolic increase with decreasing temperature. Moreover, under the freezing temperature environment, the higher the moisture content of geotextiles, the greater the puncture strength increment is.
3. Under the freezing temperature, the appearance of broken ice bodies surrounding the geotextile fibers provides a bonding force to resist tensile failure and enhances the tensile strength of the geotextiles. At low temperature, the activity of polymer molecular chains decreases and the active zone gradually shrinks, thus shortening the deformation of geotextiles.

Our methodology in this study primarily involves the application of freeze–thaw cycles to assess the durability and performance of geotextiles. These cycles simulate real-world environmental conditions, providing valuable data on the materials’ resilience. Moving forward, we plan to expand our research to include a broader range of environmental simulations, such as exposure to varying levels of humidity and ultraviolet radiation. This comprehensive approach will enable a deeper understanding of the long-term performance of geotextiles in diverse conditions, contributing significantly to the field of civil engineering materials.

## Figures and Tables

**Table 1:** Fitted results of temperature and the tensile strength of geotextiles.

Moisture Content	Fitted Equations	Correlation Coefficient
0%	Pt = −0.0033T^2^ − 0.123T + 15.339	0.996
5%	Pt = −0.0039T^2^ − 0.183T + 16.19	0.987
15%	Pt = −0.0087T^2^ − 0.062T + 16.575	0.977
30%	Pt = 0.0068T^2^ − 0.122T + 16.983	0.993
50%	Pt = 0.007^2^ − 0.112T + 17.184	0.996
80%	Pt = 0.0064T^2^ − 0.099T + 17.522	0.999

**Table 2:** Fitted results of temperature and the elongations of geotextiles at failure.

Moisture Content	Fitted Equations	Correlation Coefficient
0%	L = 27.58e0.126T	0.964
5%	L = 29.4e0.028T	0.992
15%	L = 32.18e0.026T	0.972
30%	L = 34.05e0.027T	0.951
50%	L = 36.09e0.024T	0.954
80%	L = 37.56e0.026T	0.965

**Table 3:** Fitted results of temperature and the puncture strength of geotextiles.

Moisture Content/%	Fitted Equation	R2
0%	Pp = 0.0057T^2^ − 0.0029T + 2.0809	0.9979
5%	Pp = 0.0059T^2^ − 0.0011T + 2.097	0.9982
10%	Pp = 0.0053T^2^ − 0.0041T + 2.114	0.9945
30%	Pp = 0.0053T^2^ − 0.0041T + 2.144	0.9558
50%	Pp = 0.0046T^2^ − 0.0081T + 2.137	0.9894
80%	Pp = 0.0049T^2^ − 0.0103T + 2.143	0.9842

## Data Availability

Data are contained within the article.

## References

[1] Li J., Zhang J., Yang X., Zhang A., Yu M. (2023). Monte Carlo simulations of deformation behaviour of unbound granular materials based on a real aggregate library. Int. J. Pavement Eng..

[2] Wiewel B.V., Lamoree M. (2016). Geotextile composition, application and ecotoxicology—A review. J. Hazard. Mater..

[3] Liu L., Cai G., Zhang J., Liu X., Liu K. (2020). Evaluation of engineering properties and environmental effect of recycled waste tire-sand/soil in geotechnical engineering: A compressive review. Renew. Sustain. Energy Rev..

[4] Prambauer M., Wendeler C., Weitzenböck J., Burgstaller C. (2019). Biodegradable geotextiles—An overview of existing and potential materials. Geotext. Geomembr..

[5] Correia N.d.S., Bueno B.D.S. (2011). Effect of bituminous impregnation on nonwoven geotextiles tensile and permeability properties. Geotext. Geomembranes.

[6] Wu H., Yao C., Li C., Miao M., Zhong Y., Lu Y., Liu T. (2020). Review of application and innovation of geotextiles in geotechnical engineering. Materials.

[7] Sudarsanan N., Karpurapu R., Amrithalingam V. (2018). An investigation on the interface bond strength of geosynthet-ic-reinforced asphalt concrete using Leutner shear test. Constr. Build. Mater..

[8] Liu X., Congress SS C., Cai G., Liu L., Liu S., Puppala A.J., Zhang W. (2022). Development and validation of a method to predict the soil thermal conductivity using thermal piezocone penetration testing (T-CPTU). Can. Geotech. J..

[9] Liu X., Congress SS C., Cai G., Liu L., Puppala A.J. (2022). Evaluating the thermal performance of unsaturated bentonite–sand–graphite as buffer material for waste repository using an improved prediction model. Can. Geotech. J..

[10] Saathoff F., Oumeraci H., Restall S. (2007). Australian and German experiences on the use of geotextile containers. Geotext. Geomembranes.

[11] Moo-Young H.K., A Gaffney D., Mo X. (2002). Testing procedures to assess the viability of dewatering with geotextile tubes. Geotext. Geomembranes.

[12] Kutay M.E., Aydilek A.H. (2004). Retention performance of geotextile containers confining geomaterials. Geosynth. Ternational.

[13] Hakimelahi N., Bayat M., Ajalloeian R., Nadi B. (2003). Effect of woven geotextile reinforcement on mechanical behavior of calcareous sands. Case Stud. Const. Mat..

[14] Rawal A., Sayeed M.A., Saraswat H., Shah T. (2013). A comparison of wide-width tensile strength to its axi-symmetric tensile strength of hybrid needlepunched nonwoven geotextiles. Geotext. Geomembranes.

[15] Sawicki A., Kazimierowicz-Frankowska K. (1998). Creep behaviour of geosynthetics. Geotext. Geomembr..

[16] Bacas B.M., Cañizal J., Konietzky H. (2015). Shear strength behavior of geotextile/geomembrane interfaces. J. Rock Mech. Geotech. Eng..

[17] Pinho-Lopes M., De Lurdes Lopes M. (2018). Influence of mechanical damage induced in laboratory on the soil-geosynthetic interaction in inclined-plane shear. Constr. Build. Mater..

[18] Gautier K.B., Kocher C.W., Drean J.-Y. (2007). Anisotropic mechanical behavior of nonwoven geotextiles stressed by uniaxial tension. Text. Res. J..

[19] Punetha P., Mohanty P., Samanta M. (2017). Microstructural investigation on mechanical behavior of soil-geosynthetic interface in direct shear test. Geotext. Geomembr..

[20] Kongkitkul W., Tabsombut W., Jaturapitakkul C., Tatsuoka F. (2012). Effects of temperature on the rupture strength and elastic stiffness of geogrids. Geosynth. Int..

[21] Chantachot T., Kongkitkul W., Tatsuoka F. (2018). Effects of temperature rise on load-strain-time behaviour of geogrids and simulations. Geosynth. Int..

[22] Zornberg J.G., Byler B.R., Knudsen J.W. (2004). Creep of geotextiles using time–Temperature superposition methods. J. Geotech. Geoenvironmental Eng..

[23] (2011). Standard Test Method for Tensile Properties of Geotextiles by the Wide-Width Strip Method.

[24] (2014). Standard Test Method for the Static Puncture Strength of Geotextiles and Geotextile-Related Products Using a 50-mm Probe.

[25] Hsieh J.-C., Li J.-H., Huang C.-H., Lou C.-W., Lin J.-H. (2017). Statistical analyses for tensile properties of nonwoven geotextiles at different ambient environmental temperatures. J. Ind. Text..

[26] Liu T., Yang X., Zhang Y. (2022). A Review of Gassy Sediments: Mechanical Property, Disaster Simulation and In-Situ Test. Front. Earth Sci..

[27] Liu L., Cai G., Liu S. (2018). Compression properties and micro-mechanisms of rubber-sand particle mixtures considering grain breakage. Constr. Build. Mater..

